# Menstrual cycle effects on sex differences in motor control during dynamic balance perturbations

**DOI:** 10.1113/EP093608

**Published:** 2026-03-27

**Authors:** Samuli Nevanperä, Ritva S. Mikkonen, Simon Walker, Jarmo M. Piirainen

**Affiliations:** ^1^ Sports Technology Unit Vuokatti Faculty of Sport and Health Sciences University of Jyvaskylä Vuokatti Finland; ^2^ NeuroMuscular Research Center Faculty of Sport and Health Sciences University of Jyvaskylä Jyväskylä Finland

**Keywords:** female physiology, H‐reflex, motor control

## Abstract

This study investigated sex differences in neuromuscular responses during dynamic balance perturbations and isometric maximal voluntary contractions (IMVCs) by examining if menstrual cycle (MC) phase and the fluctuations in endogenous hormones influence the observation of sex differences. Twenty‐seven young adults (17 males: 28.5 ± 6.4 years; 10 females: 31.2 ± 6.5 years) were measured twice: females were measured at the early‐follicular (EF) phase, that is 2–4 days after onset of bleeding, and at the mid‐luteal phase (LUT), coinciding with peak levels of progesterone. Males were measured twice with 7–14 days between measurements. During dynamic balance perturbations, H‐reflex and V‐wave (neural drive) responses were recorded from the posterior perturbations at four latencies (40 ms, 70 ms, 100 ms, and 130 ms) coinciding with short‐ (SLR), medium‐ (MLR), and long‐ (LLR) latency responses and voluntary activation. Further, IMVC and its associated neural drive were assessed. Sex differences in either the H‐reflex (*P *= 0.217) or the V‐wave (*P *= 0.475) were not observed during balance perturbation tasks when females at EF were compared to males. However, when the measurements were conducted at LUT, a significant sex difference was revealed in both H‐reflex (*P *= 0.029) and V‐wave (*P *= 0.049) responses. Similarly, in neural drive during IMVC, no sex differences were observed at EF (*P *= 0.440), but a significant sex difference at LUT was observed (*P *= 0.024). Our results suggest that hormonal fluctuation in two distinct MC phases lead to females having lower neural responses during dynamic balance and voluntary force generation tasks at LUT compared to males.

## Abstract (highlights)

- **What is the central question of this study?**
  Does menstrual cycle (MC) phase influence interpretation of sex differences in H‐reflex and V‐wave excitability during dynamic balance perturbations?
- **What is the main finding and its importance?**
  Whereas sex differences were not observed when males were compared to females in the early‐follicular (EF) phase, sex differences were observed when comparing males to females in the mid‐luteal phase. The H‐reflex and V‐wave during dynamic balance perturbations measured at the mid‐luteal but not at the EF phase were significantly lower in females compared to males. Observation of sex differences appears to be influenced by MC phase.

## 1 INTRODUCTION

Efficient neuromuscular function at both spinal and supraspinal levels are crucial factors for efficient balance ability and motor control (Baudry, 2016; Baudry & Duchateau, 2014; Jacobs & Horak, 2007). Although some efforts have been made to address sex differences in balance properties of young adults in static conditions (Era et al., 2006; Šarabon et al., 2022), the information regarding sex differences in motor control in younger age groups, and especially in dynamic weight‐bearing conditions, is limited. Furthermore, scientific evidence is slowly emerging to suggest that neuromuscular function may be influenced by hormonal changes associated with the menstrual cycle (MC) (Ansdell et al., 2019; Smith et al., 2002), but the data on sex differences in neuromuscular function when MC is considered remain scarce. As Mendonca et al. (2020) have indicated, the influence of MC and overall changes in ovarian hormone concentrations on sex differences in motor performance is unknown.

A natural or eumenorrhoeic MC is characterized by the fluctuation of the two main female reproductive hormones, oestradiol and progesterone. At the early‐follicular phase (EF), which begins at the onset of menses/bleeding, concentrations of oestradiol and progesterone are at their lowest. Oestradiol gradually increases during the follicular phase prior to a surge in luteinizing hormone (LH) that indicates ovulation, typically occurring at mid‐cycle. At the luteal phase, which begins after ovulation, progesterone increases reaching its peak concentration approximately 7–9 days post‐ovulation. At the mid‐luteal phase (LUT), oestradiol concentrations are higher than at the EF phase reaching a second peak prior to a concurrent decrease in oestradiol and progesterone prior to a new cycle (Casey et al., 2016; Taipale‐Mikkonen et al., 2021). In addition to reproductive function, oestradiol and progesterone are categorized as neuroactive steroids (Stoffel‐Wagner, 2001). Whereas oestradiol has been shown to have excitatory neuronal effects (Smith & Woolley, 2004; Smith et al., 2002), progesterone seems to enhance inhibitory responses, as it can influence neuronal excitability at cortical level via the inhibitory γ‐aminobutyric acid A (GABA_A_) receptor (Smith & Woolley, 2004; Stoffel‐Wagner, 2001). Originally, this GABAergic inhibition of the first‐order precursor of progesterone has been shown in rodent models (Majewska et al., 1986; Smith et al., 1989), and later studies with human participants have confirmed this (Ansdell et al., 2019; Smith et al., 2002), showing evidence of changes in cortical excitability across MC with increased inhibition and decreased corticospinal tract excitability at the mid‐luteal phase concurrent with high levels of progesterone. Thus, it can be hypothesized, that sex‐related differences in motor control may differ dependent on the concentration of endogenous hormones such as progesterone at different phases of the MC (Casamento‐Moran et al., 2017).

To examine motor control and neural mechanisms at spinal and supraspinal sites, the Hoffman reflex (H‐reflex) and volitional wave (V‐wave) can be used, respectively. The H‐reflex measures spinal level excitability and transmission efficiency from Ia afferents to motoneurons (Aagaard et al., 2002). It is elicited by applying an electrical stimulation to a peripheral nerve, which is then manifested as a monosynaptic response in muscle electromyography (EMG) (Pierrot‐Deseilligny & Mazevet, 2000). The V‐wave is an electrophysiological variant of the H‐reflex that depicts the magnitude of efferent motoneuronal output during volitional muscle contraction (Aagaard et al., 2002; Bergmann et al., 2013; Upton et al., 1971). The V‐wave can be used to measure the contribution of supraspinal activity, that is, neural drive, in motor control. For instance, Mendonca et al. (2020) studied sex differences in both H‐reflex and V‐wave responses during low‐level tonic isometric contractions. They found that females exhibited significantly a lower soleus muscle H‐reflex compared to males, but there were no significant sex differences in V‐wave amplitude, indicating no sex differences in volitional ability to activate the soleus muscle. Consistent with the findings of Mendonca et al. (2020), other studies have stated that there may not be any sex differences in the ability of central nervous system to fully activate skeletal muscles (Hunter et al., 2006, 2024).

In contrast to Mendonca et al. (2020) who reported significant sex differences in the H‐reflex during isometric tonic muscle contractions, Hoffman et al. (2018a) measured the H‐reflex from young adults in a still prone position and found no sex differences in spinal excitability. Also, Johnson et al. (2012) found no sex differences in an unconditioned H‐reflex measured in a sitting position. However, in the study by Hoffman et al. (2018b) males and females were measured in a prone position on two occasions, timing the females’ measurements in the early follicular phase and approximately 24 h after a positive ovulation test. While presynaptic inhibition measured with the H‐reflex technique showed no sex differences in the early follicular phase, females exhibited decreased presynaptic inhibition compared to males concurrent with high concentrations of oestrogen at ovulation, supporting the viewpoint that ovarian hormones may influence neuronal excitability (Stoffel‐Wagner, 2001).

The broad discrepancy in the results of these studies mentioned above may stem from the fact that very diverse methods for tracking MC and confirming hormone concentrations were used. Mendonca et al. (2020) used only self‐reported confirmation of a ‘regular’ MC and excluded individuals that were taking hormonal contraceptives. Hunter et al. (2006) only recorded the self‐reported day of the MC on which the experimental protocol was performed. In the studies by Hoffman et al. (2018a) and Johnson et al. (2012), hormonal fluctuations were controlled by measuring participants at the onset of menses/bleeding. Whereas Johnson et al. (2012) did not report any methods for verifying MC phase, Hoffman et al. (2018a) used saliva tests to report hormonal concentrations. Although these studies provide valuable insights into potential sex differences in neural mechanisms, they lacked rigorous tracking of the MC, limiting their robustness in terms of current methodological recommendations by Elliott‐Sale et al. (2021).

As Mendonca et al. (2020) stated, the influence of the MC and ovarian hormone fluctuations on sex differences in motor performance remains largely unexplored. Thus, the main purpose of our study was to determine whether the menstrual‐cycle phase in which females are tested plays a meaningful role in the observation of sex differences when compared with males. More specifically, we investigated if measurements in two hormonally distinct MC phases, EF and LUT, might influence the presence or absence of sex differences in neural responses during anterior–posterior dynamic balance perturbations and neural drive during isometric maximal voluntary contractions (IMVCs), by confirming the MC phases in line with current recommendations for best practice. To study motor control, both the H‐reflex and V‐wave were measured at four latencies corresponding with short‐latency response (SLR), medium‐latency response (MLR), long‐latency response (LLR) and voluntary activation.

## 2 METHODS

### 2.1 Ethical approval

This study was approved by the Ethics Committee of the local University (approval form reference #1087/13.00.04.00/2023). The data collection started in 2023, and thus it was conducted according to the *Declaration of Helsinki* 2013, except for registration in a database.

### 2.2 Participants

A total of 19 healthy males and 19 healthy, normally menstruating females volunteered for the study, which included two measurement sessions. Table 1 shows the main characteristics of the participants including statistical comparisons between the sexes. The inclusion criteria required that participants were between 20 and 40 years of age with no recent lower limb musculoskeletal injuries or diseases that would influence neuromuscular measurements. Females were recruited if they were not currently using or had not used hormonal contraceptives within 6 months before measurements, if they were not pregnant or lactating and had no diagnosed menstrual dysfunctions or endocrine disorders. Participants were recruited via word‐of‐mouth, social media and locally distributed posters. During the process, 19 males were recruited, of which 17 participated in both measurement sessions for test–retest analysis. With regard to females, 19 enrolled in the study, but four participants dropped out after the first measurement due to personal reasons and two participants due to an undetected LH surge. Thus, 13 females participated in both measurements. However, one participant was excluded due to violated soleus muscle EMG data, and two participants were excluded from the luteal‐phase analysis due to low levels of progesterone (<16 nmol/l) at the mid‐luteal phase in line with Elliott‐Sale et al. (2021). Thus, 10 eumenorrhoeic participants were included in data analysis.

**TABLE 1:** Characteristics (means ± SD) of males (*n* = 17) and eumenorrhoeic females (*n* = 10) with *P*‐value for sex difference.

Variables	Males (*n* = 17)	Females (*n* = 10)	*P*
Age (years)	28.5 ± 6.4	31.2 ± 6.5	0.312
Height (cm)	179.9 ± 5.2	165.0 ± 3.7	<0.001
Body mass (kg)	84.9 ± 9.1	66.1 ± 7.6	<0.001
BMI (kg/m^2^)	26.2 ± 2.4	24.3 ± 2.5	0.124

Participants received comprehensive instructions regarding the study and signed an informed consent. Prior to participation, the health and physical background questionnaire was screened. Participants were instructed to not consume caffeine on the morning of measurement, abstain from alcohol for 24 h, and ensure adequate rest by refraining from heavy lower‐body exercise for 48 h prior to measurement. Participants were aware that they were participating voluntarily, and they had the right to withdraw from the study whenever they so felt with no further consequences.

### 2.3 Study design

Both males and females were measured twice. Male participants underwent two measurements 7–14 days apart, mimicking the females’ measurement interval described below. As no significant differences were observed between measurement A and B in males, only the males’ second measurement was included in the analysis of sex differences. The second measurement was chosen to minimize any potential influence of learning or familiarization across repeated sessions. The test–retest analysis between the first (A) and second (B) male measurements are provided in the Appendix, confirming measurement stability and absence of variability.

Females were measured at two time points of the MC: (1) at the EF phase (EF; 2–4 days after first day of bleeding) and (2) at the mid‐luteal phase (LUT; 6–8 days after a positive LH‐surge). These phases were chosen to coincide with distinct hormonal milieus of (1) low oestradiol and low progesterone and (2) high oestradiol and high progesterone, respectively. The first measurement for females was randomized to reduce the possible influence of a learning effect (5/10 started at EF and 5/10 at LUT). Participants used a urine‐based ovulation kit (The Clearblue® Advanced Digital Ovulation Test, Swiss Precision Diagnostics GmbH, Geneva, Switzerland) to detect the surge of LH, which is an indicator of ovulation. The use of the Clearblue® ovulation kit was instructed verbally and/or by instruction leaflet.

For both males and females, measurements were conducted at the same time of day to avoid the influence of circadian rhythm (Beníčková et al., 2025). Each testing session began with Inbody test (InBody 770 body composition analyser; InBody, Cerritos, CA, USA) and venous blood sample taken in a fasted state between 08.00 and 10.00 h. After that, breakfast was served and the participants continued to motor control measurements. The measurement sessions consisted of familiarization (only at the beginning of first measurement), surface EMG electrode preparations, 8‐min warm‐up with 80 W with Monark LC7TT‐cycle ergometer (Monark, Vansbo, Sweden), H‐reflex recruitment curve, isometric maximal force production and neural drive measurements in isometric condition, and finally dynamic balance perturbation tasks in anterior–posterior direction with and without electrical stimulations (H‐reflex and V‐wave). Measurement protocols were identical for females and males.

### 2.4 Serum hormones

Venous blood samples were taken in the morning between 08.00 and 10.00 h in a fasted state by a qualified nurse. Blood samples were collected into two serum tubes (Vacuette, Greiner Bio‐One, Kremsmünster, Austria). The samples were centrifuged with Thermo Scientific Medifuge (Thermo Electron LED GmbH, Osterode am Harz, Germany) with Relative Centrifugal Force (RCF) of 2100 g for 15 min, pipetted into 1.5 and 2 mL Eppendorf tubes (Sarstedt AG & Co. KG, Nümbrecht, Germany) and serum was stored in the freezer at −80°C until further analysis of total testosterone (tTesto in nmol/l) for males and oestradiol (E2 in pmol/l) and progesterone (P4 in nmol/l) and the E2:P4 ratio for females. E2:P4 was reported as the pmol‐to‐nmol ratio. After analysis, if the P4‐concentrations at LUT was <16 nmol/l, the participant was excluded from data analysis in line with (Elliott‐Sale et al., 2021).

### 2.5 Familiarization

Familiarization was carried out only at the beginning of the first measurement session. The participant was familiarized with electrical stimulation, dynamic balance perturbations and isometric force production. The custom‐built isometric dynamometer (University of Jyväskylä, Jyväskylä, Finland) was adjusted and the distance of the bench from the force plate was recorded to a measurement log to ensure the same settings for both measurements. Participants were instructed to perform with proper technique ensuring efficient force production as well as proper and desired posture prior to dynamic balance perturbations. The familiarization protocol of the dynamic balance perturbations included one additional set of 20 perturbations prior to actual measurements to habituate participants to sudden perturbations.

### 2.6 Surface electromyography and H‐reflex recruitment curve

The surface EMG‐electrodes (Blue Sensor, Ag/AgCl, ⌀ 28 mm, Ambu A/S, Ballerup, Denmark) were placed bipolarly to the soleus (SOL) and tibialis anterior (TIB) muscles of the right leg with an interelectrode distance of 2 cm. EMG‐electrodes on the SOL were placed in line with the Achilles tendon approximately 4 cm below the medial gastrocnemius, which was carefully palpated to avoid cross talk, which could have interfered with SOL muscle EMG. TIB was palpated, and the EMG‐electrodes were placed on the belly of the muscle, according to SENIAM instructions (Hermens et al., 1999). Before placing the electrodes, the skin was prepared according to SENIAM instructions (Hermens et al., 1999): skin was gently abraded with fine sandpaper and cleaned with alcohol. After placing the electrodes, the required impedance of <5 kΩ between the skin and the electrodes was checked and thereafter the electrodes were taped to the skin with sports tape (Leukoplast, Hamburg, Germany) to avoid possible movement of the electrodes.

To measure the maximal M‐wave and H‐reflex of SOL‐muscle, the H‐reflex recruitment curve was implemented in a quiet standing position using a Digitimer stimulator (Constant current stimulator DS7R, Digimeter Ltd, Welwyn Garden City, UK) with 200 µs square pulses. An oval‐shaped anode (5 × 8 cm) was placed over the patella. The best possible stimulation spot (highest M‐wave with constant low‐intensity stimulation) was observed by localizing the tibial nerve travelling in the popliteal fossa by using a temporary cathode and with constant stimulation intensity. Thereafter, an EMG‐electrode (Blue Sensor, Ag/AgCl, ⌀ 28 mm, Ambu A/S, Ballerup, Denmark) was placed on the desired spot and taped firmly with an elastic bandage rolled over the knee joint to avoid electrode movement. This setting remained throughout the entire measurement session. To determine the maximal H‐reflex and maximal M‐wave (*M*_max_), stimulations were delivered at 8‐s intervals, starting with a very low intensity and increased by 1 mA until the maximal H‐reflex was found. After that, the stimulation intensity was increased by approximately 5 mA steps until *M*_max_ was reached (Gajewski & Mazur‐Rożycka, 2016). Finally, to ensure that the maximal M‐wave had been reached, two to four supramaximal (150% of *M*_max_) stimulations were delivered. The EMG signal (muscle activity) and stimulation responses were collected using a Neurolog system (NeuroLog NL900D; Hertfordshire, UK; Digitimer), amplified 1000×, band‐bass filtered to 15–500 Hz and the sampling rate was set 1 kHz with CED analog‐to‐digital converter (CED Power 1401; CED Ltd, Cambridge, UK). The data were recorded with Spike2 8.11‐software (CED).

### 2.7 Isometric maximal voluntary contraction and neural drive measurements

**FIGURE 1:**
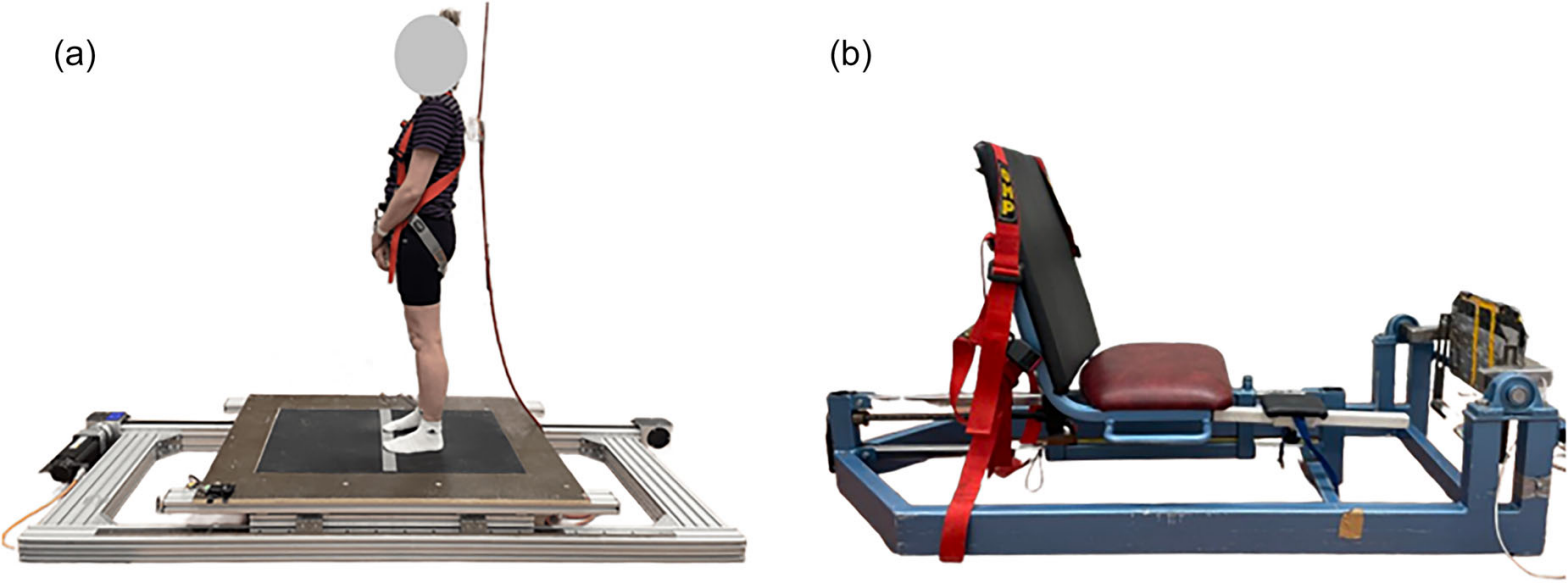
(a) The custom‐built dynamic balance perturbation device used in this study. The device induced 20 perturbations in one set in anterior–posterior direction in a randomized order. A custom‐built force plate was mounted in the middle under a black gripped mat. The participant was secured with harness and instructed to stand still with their feet straight in the middle of the plate and with their hands in front them. (b) The custom‐built isometric dynamometer, which was used in IMVC measurements.

Isometric maximal voluntary contraction (IMVC) was conducted using the custom‐built isometric dynamometer (University of Jyväskylä, Jyväskylä, Finland) (Figure 1b) producing plantar flexion only with the right leg. The participant sat on the bench with their back firmly against the bench in a position of hip, knee and ankle angle of 110°, 180° and 90°, respectively (knee fully extended). Participants performed a warm‐up, which consisted of 2 × 10 submaximal force productions between 50% and 90% of subjective maximal effort. The actual IMVC measurements consisted of three trials of 3 s maximal force productions with 1‐min intervals. The participant was instructed to hold on to the grips with their hands, to set their right foot against the platform to the same place with each performed contraction, to take a deep breath just prior to the go‐signal (3‐2‐1, Go!), and produce force as fast as possible and push all‐out until 3 s had elapsed, and the researcher stopped cheering. If a clear improvement (>5%) was observed on the third trial compared to the second best, one to two extra trials were executed to ensure that the maximal force levels had been achieved. The measurements of maximal tibialis anterior (TIB) muscle EMG activity (as an agonist) with dorsi flexion were implemented in the same position as in IMVC with the same three trials of 3 s maximal force production with 1 min of rest. Participants were instructed to produce a dorsi flexion against the researcher's hand, as the researcher held on from the participants toes and produced a counterforce. Due to the limitation of the device (only plantar flexion force), no force data were obtained for TIB, but only EMG data.

Next, neural drive (V‐wave) was measured from the right leg SOL muscle during IMVC according to (Nevanperä et al., 2023). A total of five successful trials had to be completed. The trial was judged as successful if: (1) the participant reached at least the 90% force level, (2) stimulation was timed and elicited in an ascending/plateaued force curve after crossing the 90% force limit, (3) the M‐wave was maximal and (4) the V‐wave was visually noticeable from the computer screen. Participants performed a total of five to eight trials with 1‐min intervals to achieve the required five successful trials.

### 2.8 Dynamic balance perturbations

A custom‐built dynamic balance platform (University of Jyväskylä, Vuokatti, Finland) (Figure 1a) was used to induce perturbations. A custom‐built force plate (University of Jyväskylä) was mounted on top of the platform, and the dimensions of the force plate were 100 cm × 65 cm × 1.5 cm, and the total length and width of the device was 282 and 105 cm, respectively. The platform was operated from the controlling unit (IndraDrive Cs, Bosch Rexroth, Lohr am Main, Germany) using a servomotor (Bosch Rexroth, 3‐phase synchronous pm‐motor), which produced perturbations via a motor‐driven belt with an acceleration of 2.5 m/s^2^, maximum velocity 0.25 mm/s and displacement of 0.3 m in anterior–posterior direction, executed by LabVIEW (National Instruments, Austin, TX, USA) and IndraWorks (Bosch Rexroth) software.

Twenty perturbations in both anterior (*n* = 10) and posterior (*n* = 10) directions were executed in a randomized order, which allowed the platform to move a maximum of two perturbations in a row to the same direction. The order was set so that the platform returned to its original (middle) position after the last trial of the set. The platform movement at every trial was only triggered if the COP was inside ±5 mm from the zero level for at least 1 s. This ensured that the participant was in a stable condition and standing straight without any anticipation of the upcoming perturbation. The balance data were collected by Coachtech‐software (University of Jyväskylä) with a sampling rate of 400 Hz. Raw force signals were low pass filtered using Finite Impulse Response (FIR) filters with 25 Hz as cut‐off frequency.

During the dynamic balance perturbation, the participants wore a safety harness (CAMP Empire, Perth, Australia) to ensure they would not fall and injure themselves. The harness was adjusted to secure the participant, but not to support the participant during the perturbation. Participants were instructed to stand straight with their hands in front of them (see Figure 1a) and stabilize the vision to a green dot on the wall at 3 m distance. Further, the participants were instructed to react to the upcoming perturbations in a natural manner, as efficiently as the situation demands. Just prior to actual measurements with electrical stimulation, the participant performed one additional familiarization set of 20 perturbations to minimize the learning effect, ease anxiety and reduce excessive stress‐induced muscle tension and muscle activity.

Both H‐reflex and V‐wave recordings were implemented at four different latencies from the onset of ankle movement. According to our previous study, the latency between the onset of trigger signal and the ankle movement was constant 25 ms (Hu et al., 2022). The latencies were determined according to literature to coincide with the SLR (Corden et al., 2000), MLR (Miranda et al., 2019), LLR (Miranda et al., 2019) and voluntary activation (Taube et al., 2008). Consequently, the latencies were set to 40, 70, 100 and 130 ms, respectively, from the onset of ankle movement. During the H‐reflex recordings, the M‐wave was set to 10 ± 3% of *M*_max_ (measured in standing rest during the H‐reflex recruitment curve) to keep the H‐reflex on an ascending curve (Knikou, 2008). A Spike2 script was used online during the measurements to follow and control the amplitude of the M‐wave. Consequently, only those trials of posterior perturbations (participant swaying forward) where the M‐wave stayed inside the determined percentual level of 10 ± 3% of *M*_max_ were chosen to further analysis. At least eight successful trials were required, and if this was not achieved in one set, an extra set was performed. During the V‐wave measurements, the same four latencies (40, 70, 100 and 130 ms) were measured with a supramaximal stimulation intensity as used in the V‐wave recordings during IMVC. Finally, one set of 20 perturbations was executed without electrical stimulation to collect background muscle activity and balance properties. The protocol was built so that the H‐reflex was always measured first, the V‐wave second and the set with no stimulation last. However, the order of execution between latencies was randomized between participants but kept the same for an individual at both measurement sessions. Thus, a total of 180–260 perturbations were induced (with a 2‐min sitting break after every third set) during the dynamic balance perturbations, depending on whether extra sets were needed in H‐reflex recordings.

### 2.9 Data analysis

From the H‐reflex recruitment curve, the *M*_max_ was recorded, as well as the latencies of the coincidence of both H‐reflex and M‐wave responses according to stimulation artifact, which were later used in an on‐line script measuring the neural responses during the dynamic balance perturbations.

From the IMVC data, the maximum force in newtons (N) was measured. The peak‐to‐peak V‐wave during IMVC was analysed and normalized to concurrent maximal M‐wave in each trial. The V‐wave/M‐wave ratio of each trial was calculated and consequently the five successful trials were averaged and reported as *V*_iso_/*M*_max_%. The maximal EMG activity of both SOL and TIB was analysed from the IMVC trials and determined as root mean square (RMS) over an epoch of 500 ms. From SOL, the maximal EMG activity was analysed from the best trial (highest force level) by sweeping through the plateaued force phase. As for TIB, no force was measured, and thus all three trials of muscle EMG were swept through to find the highest RMS value for a 500 ms time window. However, the maximal EMG activity of both SOL and TIB was used only for normalization and was not reported.

During dynamic balance perturbations both peak‐to‐peak H‐reflex and peak‐to‐peak V‐wave responses were analysed from each latency (40, 70, 100 and 130 ms) of posterior perturbations. Consequently, the averages of all successful trials (*n* = 8–16) within each separate latency were averaged for each participant and presented as *H*_bal_/*M*_max_%. The V‐wave was also analysed only from the posterior perturbations (*n* = 10) and from each latency. However, the V‐wave was normalized to concurrent maximal M‐wave, and all 10 posterior trials were averaged for each latency and for each participant and presented as *V*_bal_/*M*_max_%. The repeatability of the H‐reflex and V‐wave responses during dynamic balance perturbations has been reported elsewhere (Nevanperä et al., 2023).

Further, the last set with no stimulation was used for recording both balance properties and background EMG, which were also analysed only from the posterior perturbations (*n* = 10). Background muscle EMG was measured from both SOL and TIB muscles over a continuous 300 ms time window from the onset of ankle movement. Both SOL and TIB background activity were rectified and normalized to maximal EMG activity obtained during IMVC and multiplied by 100 to express the results as percentage. Next, to depict the reciprocal inhibition and the amount of co‐activation, the TIB/SOL ratio was calculated and multiplied by 100 to express the results as a percentage. Each outcome (SOL and TIB background activity and the TIB/SOL ratio) during dynamic balance perturbations in posterior direction were reported in 20 ms time windows, ±10 ms (Nevanperä et al., 2023) of the stimulation onsets (40, 70, 100 and 130 ms) and reported as SOL_EMG_20_ _ms_%, TIB_EMG_20_ _ms_% and TIB/SOL_RATIO_%.

The analysed balance parameters were derived from the changes in centre‐of‐pressure (COP) and calculated by the Coachtech‐software (University of Jyväskylä). From the posterior perturbations, two anatomically defined directions of COP were analysed: anterior–posterior directional sway and mediolateral directional sway. Balance parameters were analysed from the onset of platform movement until it stopped (giving an epoch of 1.2 s) by COP peak‐to‐peak displacement as millimetres (COP_p‐to‐p_), COP total trace in millimetres (COP_total trace_) and swaying velocity as mm/s (COP_velocity_). All the posterior trials were averaged and finally normalized to individual value of height × body mass (Chiari et al., 2002) and multiplied by 100 to have more representative values.

### 2.10 Statistical analysis

IBM SPSS Statistics for Windows 26.0 (IBM Corp., Armonk, NY, USA) was used for statistical analysis and result visualizations were implemented with GraphPad Prism software version 9.1.0 (GraphPad Software, Boston, MA, USA).

The normality of data were analysed with Shapiro–Wilk test of normality. For hormones and dynamic balance parameters and data obtained from isometric maximal voluntary contractions (IMVCs), an independent samples Student's *t*‐test was applied. When the assumption of normality was violated, the nonparametric Mann–Whitney *U*‐test was used instead. The data are represented as means ± SD. For *H*_bal_/*M*_max_%, *V*_bal_/*M*_max_%, SOL_EMG_20_ _ms_%, TIB_EMG_20_ _ms_% and TIB/SOL_RATIO_%, a linear mixed model (LMM) was used to examine changes in the dependent variables across latencies and to test whether these changes differed between sexes. Prior to analyses, the distribution of the data was evaluated for normality. Visual inspection of histograms revealed that the data of *V*_bal_/*M*_max_%, SOL_EMG_20_ _ms_% TIB_EMG_20_ _ms_% and TIB/SOL_RATIO_% were not normally distributed and exhibited negative skewness (skewed to the left). To address this violation of the assumption of normality, a square root transformation was applied to these data. This transformation improved the symmetry of the distribution and reduced skewness, thereby making the data more consistent with the assumptions underlying parametric testing. All subsequent analyses were performed on the transformed data but reported in the Results as original values.

The LMM model included sex, latency and their interaction (sex × latency) as fixed effects. Participant ID was treated as a random subject factor, and repeated measurements across latencies were modelled using a first order autoregressive (AR(1)) covariance structure. Model parameters were estimated using restricted maximum likelihood (REML), and Satterthwaite's method was applied to compute denominator degrees of freedom. Estimated marginal means were derived for sex, latency and the sex × latency interaction. Pairwise comparisons were performed with LSD adjustment.

In statistical analysis where females as one group were compared to males (age and body composition characteristics depicted in Table 1) and where only females were analysed (endogenous hormone concentrations between EF and LUT), the level of significance was set at *P *< 0.05. As other statistical comparisons were made for males twice (Males vs. Females at EF and Males vs. Females at LUT), the possibility for Type I error was addressed by using the Bonferroni correction. Thus, for these statistical tests the significance was set at *P* ≤ 0.025.

## 3 RESULTS

Significant differences in endogenous hormone concentrations were observed between EF and LUT. E2 concentrations were significantly higher at LUT compared to EF (at EF: 113.2 ± 72.9 pmol/l; at LUT: 487.3 ± 145.8 pmol/l; *P *< 0.001). Similarly, P4 concentrations were significantly higher at LUT compared to EF (at EF: 1.0 ± 0.3 nmol/l; at LUT: 30.9 ± 8.2 nmol/l; *P *< 0.001). The E2:P4 ratio showed a significant decrease from EF to LUT, with mean ± SD of 110.8 ± 48.6 at EF and 17.4 ± 8.0 at LUT (*P *< 0.001).

### 3.1 Neuromuscular measurements during dynamic balance perturbations

#### 3.1.1 Sex differences in H‐reflex responses

**FIGURE 2:**
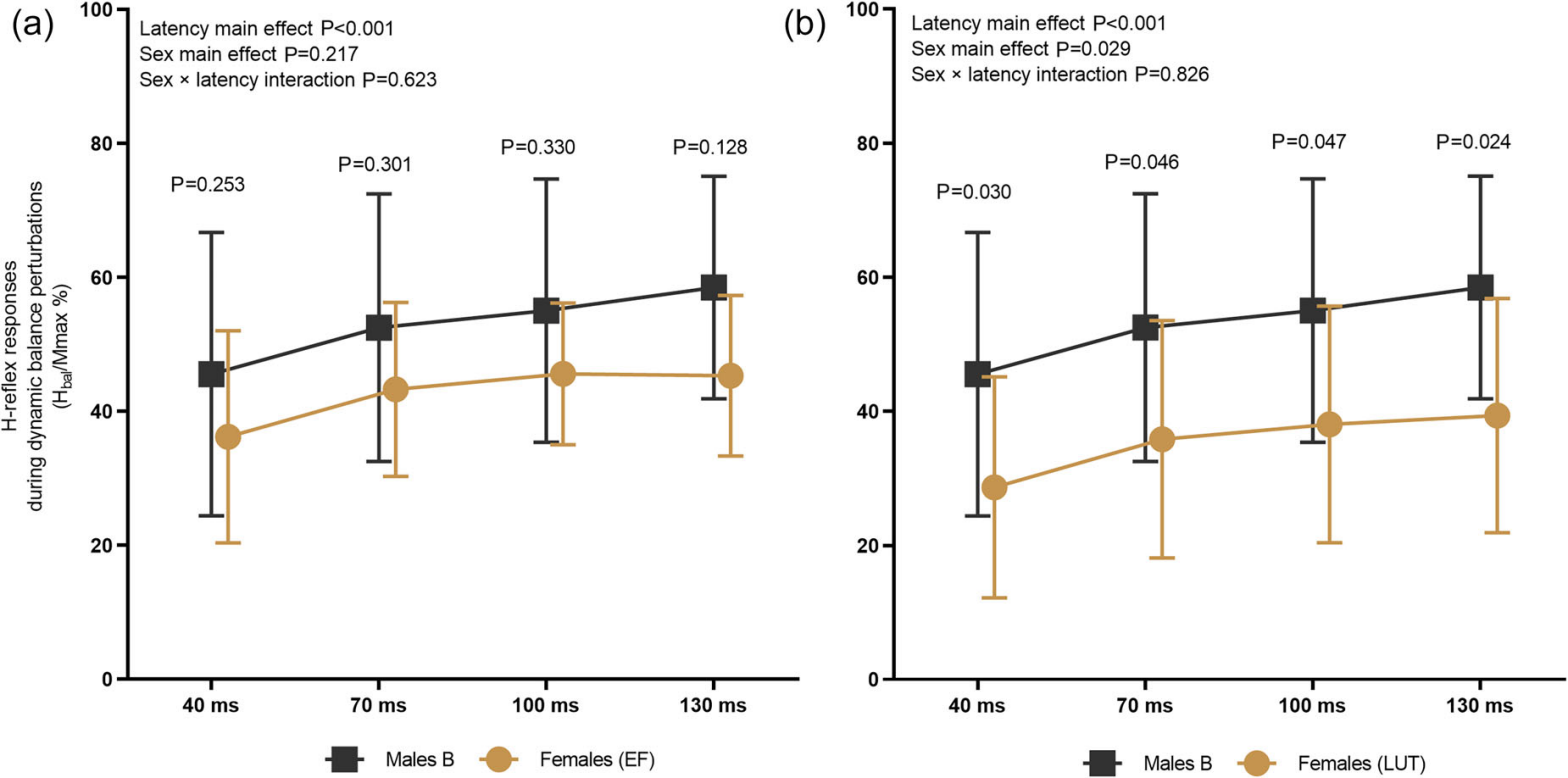
H‐reflex normalized to *M*_max_ standing rest (*H*_bal_/*M*_max_%) depicted throughout the balance perturbation at four latencies (40, 70, 100 and 130 ms) from the onset of ankle movement. Males are compared to females at EF (a) and females at LUT (b). Notice the significant sex main effect when comparing males to females at LUT.

When comparing males to females at EF (Figure 2a), no significant sex × latency interaction [*F*(3, 71.140) = 0.591, *P *= 0.623], nor sex main effect [*F*(1, 24.562) = 1.607, *P *= 0.217] was observed in *H*_bal_/*M*_max_%. Nevertheless, a significant latency main effect was observed [*F*(3, 71.140) = 9.514, *P *< 0.001]. Pairwise comparison did not reveal any significant sex differences at latencies of 40, 70, 100 and 130 ms. When comparing males to females at LUT (Figure 2b), no sex × latency interaction was observed [*F*(3, 74.477) = 0.299, *P *= 0.826], but a significant sex main effect was revealed [*F*(1, 25.610) = 5.325, *P *= 0.029]. Also in this comparison, a significant latency main effect was observed [*F*(3, 74.477) = 11.048, *P *< 0.001]. Pairwise comparison showed significant sex difference at all latencies: 40 ms (*P *= 0.030), 70 ms (*P *= 0.046), 100 ms (*P *= 0.047) and 130 ms (*P *= 0.024).

#### 3.1.2 Sex differences in V‐wave responses

**FIGURE 3:**
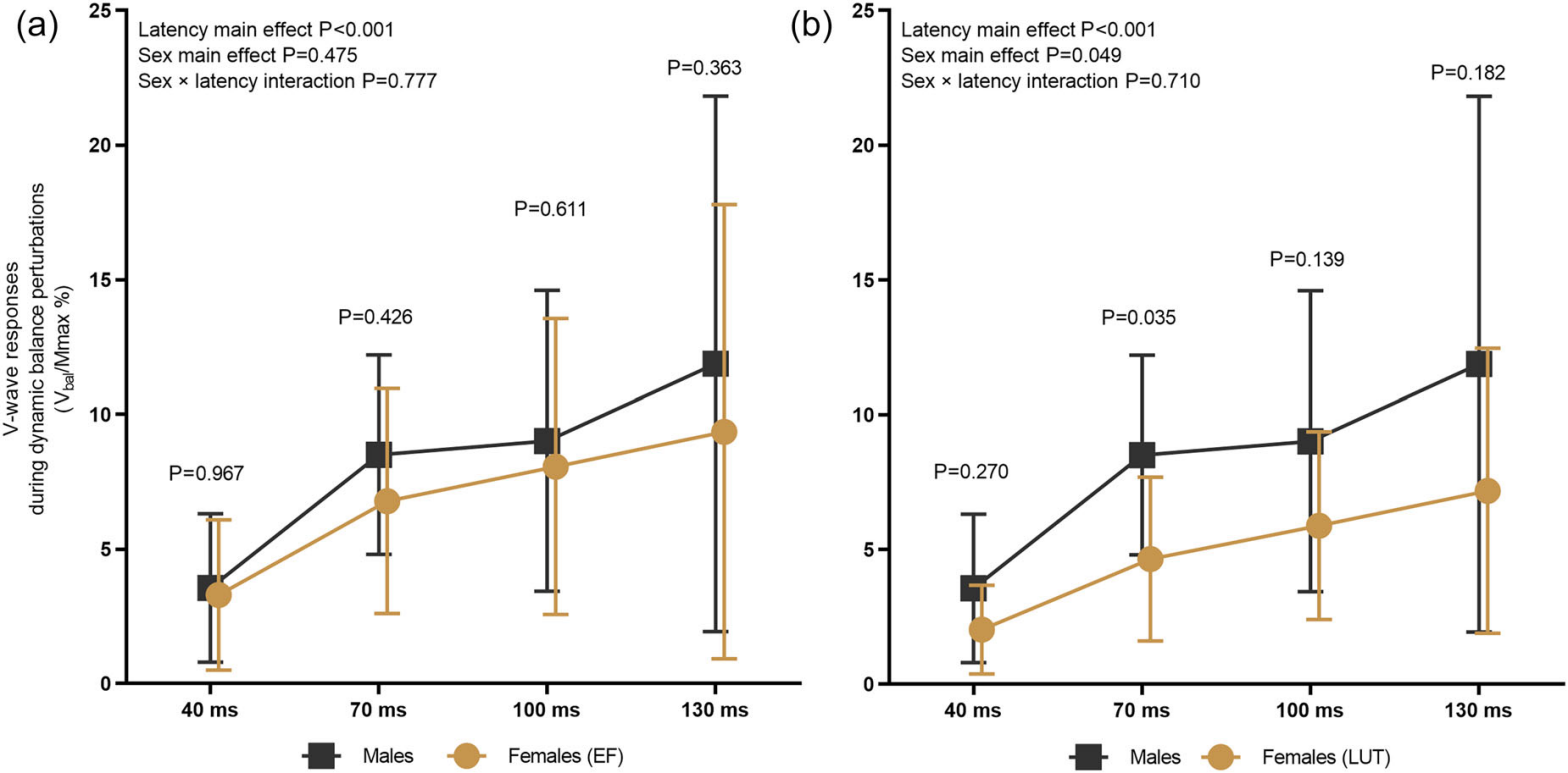
Neural drive measured with V‐wave and normalized to *M*_max_ standing rest (*V*_bal_/*M*_max_%) depicted throughout the balance perturbation at four latencies (40, 70, 100 and 130 ms) from the onset of ankle movement. Males are compared to females at EF (a) and LUT (b).

When comparing males with females at EF (Figure 3a), neural drive measured as the V‐wave during dynamic balance perturbations showed no significant sex × latency interaction [*F*(3, 64.200) = 0.367, *P *= 0.777] nor sex main effect [*F*(1, 22.727) = 0.527, *P *= 0.475], but a significant latency main effect was observed [*F*(3, 64.200) = 11.362, *P *< 0.001]. No significant sex differences were observed in pairwise comparisons of each latency. When comparing males with females at LUT (Figure 3b), no sex × latency interaction was observed [*F*(3, 63.400) = 0.462, *P *= 0.710]. However, also here a statistically significant sex main effect was observed [*F*(1, 23.400) = 4.331, *P *= 0.049] as well as a significant latency main effect [*F*(3, 63.400) = 12.531, *P *< 0.001]. Pairwise comparison revealed significant sex difference only at latency of 70 ms (*P* = 0.035).

#### 3.1.3 Muscle background EMG activity and agonist–antagonist co‐activation

During dynamic balance perturbations in SOL_EMG_20_ _ms_% between males and females at EF, no significant sex × latency interaction [*F*(3, 63.038) = 0.604, *P *= 0.615] nor sex main effect [*F*(1, 23.712) = 0.632, *P *= 0.434] was observed. However, a significant latency main effect was observed [*F*(3, 63.038) = 15.876, *P *< 0.001]. The same was the case when comparing males with females at LUT: no sex × latency interaction [*F*(3, 59.844) = 0.576, *P *= 0.633] nor sex main effect [*F*(1, 22.787) = 0.440, *P *= 0.514] was observed but a significant latency main effect was observed [*F*(3, 59.844) = 15.294, *P *< 0.001].

When examining the background activity of TIB_EMG_20_ _ms_% between males and females at EF, a significant sex × latency interaction was observed [*F*(3, 61.364) = 3.336, *P *= 0.025], but no significant sex main effect [*F*(1, 22.087) = 2.819, *P *= 0.107]. A significant latency main effect [*F*(3, 61.364) = 31.457, *P *< 0.001] was also found. When examining the pairwise comparisons, females showed a higher level of TIB activity at each 20 ms time window coinciding with the stimulation latencies, although this result was significant only in the last time window: 30–50 ms: males 0.73 ± 0.39%, females 0.85 ± 0.22% (*P *= 0.490); 60–80 ms: males 0.85 ± 0.39%, females 1.13 ± 0.43% (*P *= 0.238); 90–110 ms: males 1.67 ± 0.93%, females 1.82 ± 0.61% (*P *= 0.545); and 120–140 ms: males 2.03 ± 1.19%, females 3.29 ± 1.57% (*P *= 0.005). However, when comparing males and females at LUT, no significant results were observed in sex × latency interaction [*F*(3, 61.898) = 2.054, *P *= 0.116] nor in sex main effect [*F*(1, 21.230) = 0.895, *P *= 0.355]. Again here, a significant latency main effect was observed [*F*(3, 61.898) = 30.548, *P *< 0.001)].

**FIGURE 4:**
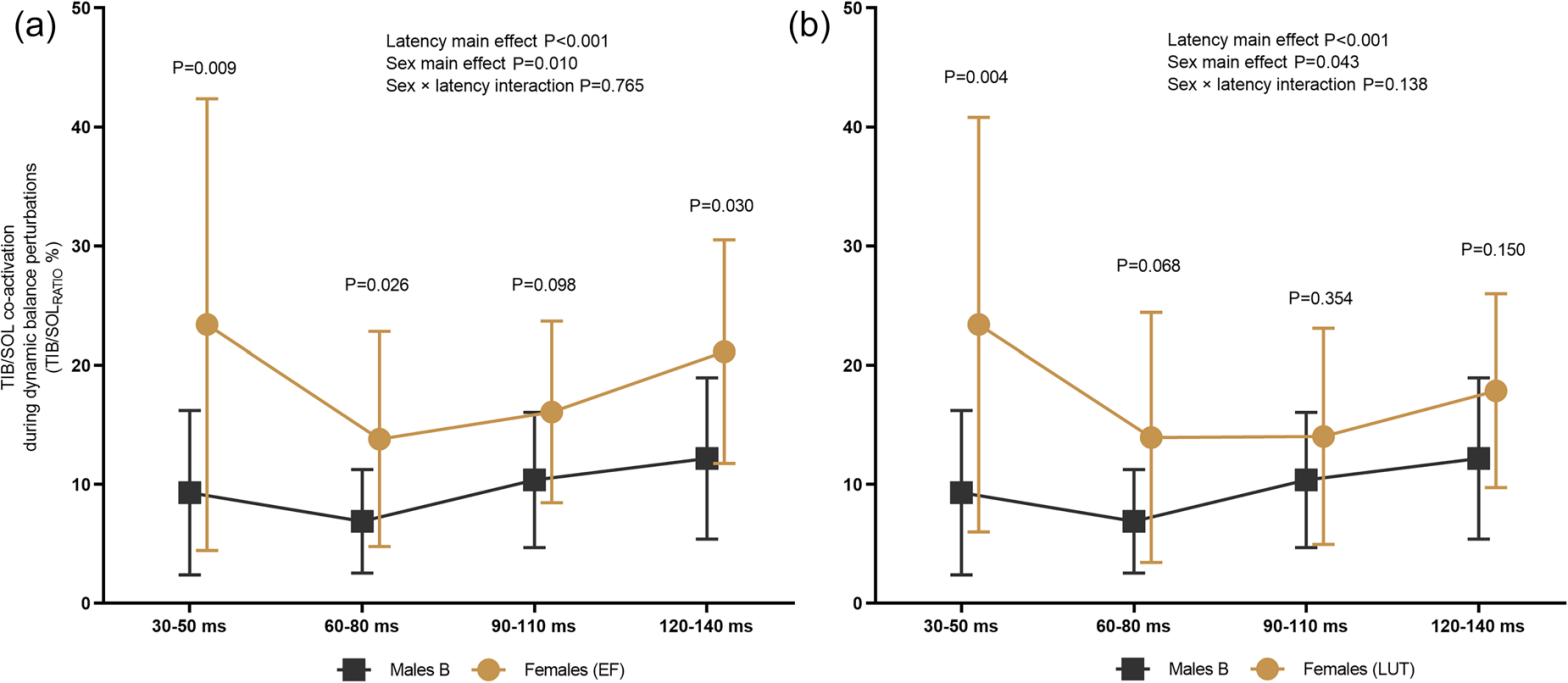
TIB/SOL_RATIO_% expressed as percentage. Males are compared to females at EF (a) and LUT (b). The TIB/SOL_RATIO_% was measured from the set of perturbations with no stimulations given and calculated in 20 ms time windows, ±10 ms, coinciding with stimulation latencies used in this study.

TIB/SOL_RATIO_% showed significant sex difference between males and females either at EF (Figure 4a) or at LUT (Figure 4b). Between males and females at EF, no significant sex × latency interaction was observed [*F*(3, 55.046) = 0.383, *P *= 0.765]. However, a significant sex main effect [*F*(1, 19.993) = 7.994, *P *= 0.010] as well as latency main effect [*F*(3, 55.046) = 4.269, *P *< 0.001] was observed. Pairwise comparison revealed significant sex differences at time windows of 30–50 ms (*P *= 0.009), 60–80 ms (*P *= 0.026) and 120–140 ms (*P *= 0.030). Similarly, when comparing males and females at LUT, no significant sex × latency interaction [*F*(3, 56.930) = 1.914, *P *= 0.138] was observed, but a significant sex main effect [*F*(1, 20.193) = 4.653, *P *= 0.043] was found as well as significant latency main effect [*F*(3, 56.930) = 6.858, *P *< 0.001]. A pairwise comparison revealed a significant sex difference only at the first time window of 30–50 ms (*P *= 0.004).

### 3.2 Isometric maximal voluntary contraction and neural drive

**FIGURE 5:**
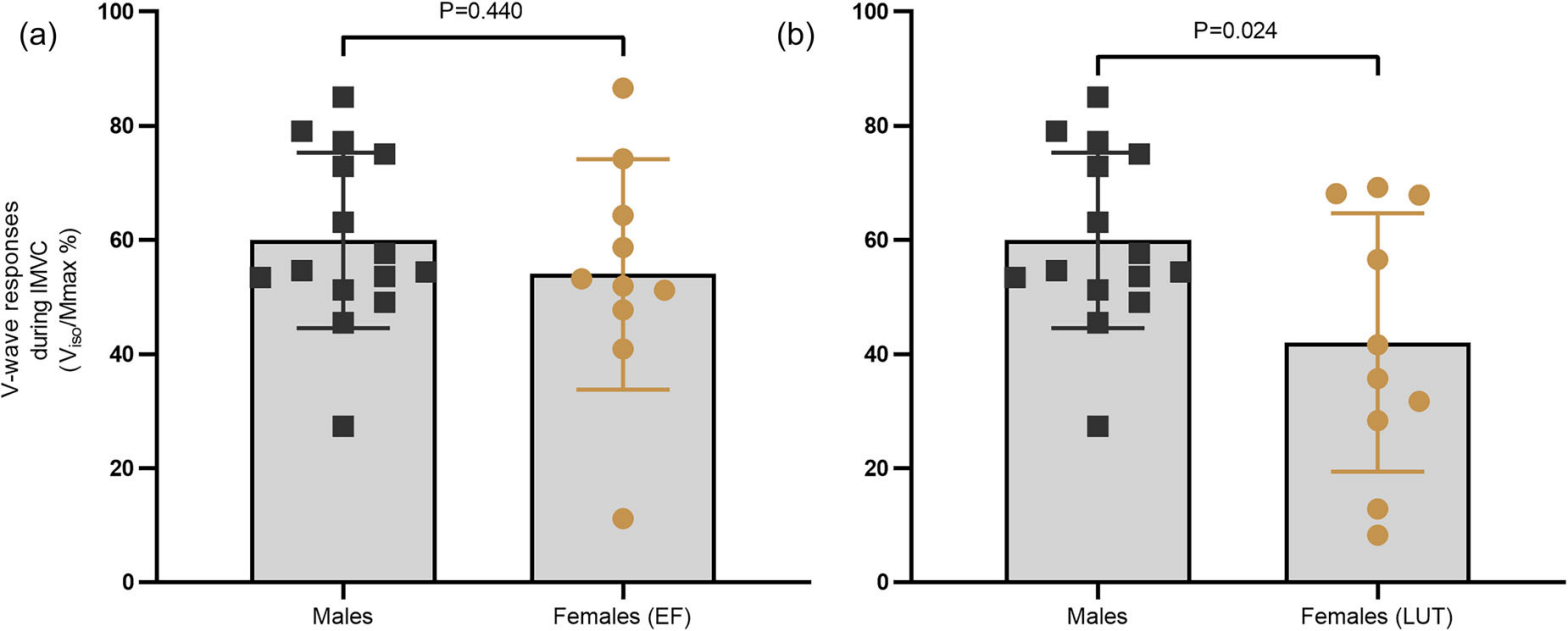
Neural drive depicted as *V*_iso_/*M*_max_% between males and females at EF (a) and males and females at LUT (b). Notice the significant sex differences when females were measured at LUT in (b).

A significant sex difference in IMVC was observed when comparing males with females at both EF (*P *< 0.001) and LUT (*P *< 0.001). Results for IMVC are presented in Table 2. Neural drive measurements during IMVC showed similar levels of *V*_iso_/*M*_max_% between males and females at EF (*V*_iso_/*M*_max_%: males 54.7 ± 20.0%; females at EF 54.0 ± 19.1%; *P *= 0.440) (Figure 5a). However, neural drive was significantly lower at LUT compared to males (Figure 5b) (*V*_iso_/*M*_max_%: males 54.7 ± 20.0%; females at LUT 42.0 ± 21.5%; *P *= 0.024).

**TABLE 2:** Results from isometric maximal voluntary contraction of plantar flexion as well as dynamic balance from anterior–posterior sway and mediolateral sway.

Variables	Males	Females (EF/LUT)		*P*
Isometric maximal voluntary contraction				
IMVC (N)	2335.4 ± 373.2	EF	1807.6 ± 212.7	<0.001
		LUT	1829.6 ± 258.8	<0.001
Dynamic balance parameters				
Anterior‐posterior direction sway				
COP_p‐to‐p_ (mm/(weight × height))	0.950 ± 0.261	EF	1.432 ± 0.554	0.018
		LUT	1.423 ± 0.593	0.021
COP_velocity_ (mm s^−^^1^/(weight × height))	1.144 ± 0.372	EF	1.641 ± 0.608	0.016
		LUT	1.675 ± 0.760	0.025
COP_total trace_ (mm/(weight × height))	1.779 ± 0.570	EF	2.573 ± 0.916	0.012
		LUT	2.637 ± 1.160	0.018
Mediolateral direction sway				
COP_p‐to‐p_ (mm/(weight × height))	0.744 ± 0.736	EF	1.143 ± 1.072	0.272
		LUT	1.088 ± 1.224	0.714
COP_velocity_ (mm s^−^^1^/(weight × height))	1.144 ± 1.192	EF	2.514 ± 2.491	0.105
		LUT	2.417 ± 2.745	0.288
COP_total trace_ (mm/(weight × height))	1.681 ± 1.841	EF	3.926 ± 2.458	0.152
		LUT	3.130 ± 3.321	0.308

*Note*: Data are Means ± SD. Results include comparison of males to females at the early‐follicular phase (EF) and mid‐luteal phase (LUT). All values of dynamic balance parameter are normalized to weight × height. Level of significance set at *P* ≤ 0.025. Abbreviations: IMVC, isometric maximal voluntary contraction of plantar flexion; COP_p‐to‐p_, centre of pressure in peak‐to‐peak amplitude; COP_velocity_, centre of pressure in velocity; COP_total trace_, centre of pressure in total sway.

### 3.3 Dynamic balance properties

#### 3.3.1 Anterior‐posterior direction sway

During dynamic balance perturbations the COP in the anterior–posterior direction showed significant sex differences regardless of MC phase. Females exhibited significantly larger normalized (weight and height) swaying at both EF and LUT in all parameters than males: COP_p‐to‐p_; *P *= 0.018 and *P *= 0.021, respectively, COP_velocity_; *P *= 0.016 and *P *= 0.025, respectively and COP_total trace_; *P *= 0.012 and *P *= 0.018, respectively (table 2).

#### 3.3.2 Mediolateral direction sway

The COP in the mediolateral direction revealed no sex differences when males were compared to females at EF and LUT. COP_p‐to‐p_; *P *= 0.272 and *P *= 0.714, respectively, COP_velocity_; *P *= 0.105 and *P *= 0.288 and COP_total trace_; *P *= 0.152 and *P *= 0.308, respectively (table 2).

## 4 DISCUSSION

This study examined sex differences in motor control and muscle co‐activation occurring during dynamic balance perturbations as well as neural drive during IMVCs in young adults. Specifically, we investigated if measurements in two hormonally distinct MC phases, EF and LUT, might influence the observation of sex differences in motor control during anterior–posterior dynamic balance perturbations.

Our results suggest that MC phase may influence observations of sex differences. Sex differences in neural responses were not observable when females at EF were compared to males, but the measurements conducted at LUT – characterized by higher levels of oestrogen and progesterone than EF – revealed significant sex differences. Previously, sex differences in cortically mediated neural activation have not been reported (Hunter et al., 2006; Mendonca et al., 2020); however, these studies have not confirmed menstrual status nor MC phase of participants. Indeed, the present study revealed sex differences in maximal neural drive during IMVC, showing significantly lower *V*/*M*_max_ in females at LUT compared to males – a difference not observed at EF. These results suggest that hormonal concentrations associated with MC phase may influence our interpretation of the presence or absence of sex differences in neural mechanisms.

Several earlier studies support these findings, indicating that both oestrogen and progesterone act as neurosteroids capable of crossing the blood–brain barrier and influencing neuronal excitability (Stoffel‐Wagner, 2001). Furthermore, high oestrogen levels have been associated with decreased presynaptic inhibition at ovulation (Hoffman et al., 2018b) and excitatory neuronal effects at the late‐follicular phase (Smith et al., 2002), whereas progesterone appears to exert inhibitory neuronal effects. This non‐reproductive inhibitory action of progesterone is mediated through the γ‐aminobutyric acid A (GABA_A_) receptor (Ansdell et al., 2019; Callachan et al., 1987). Ansdell et al. (2019) measured females at three distinct MC phases and showed that hormonal fluctuations influenced central nervous system function and motor performance. They drew the conclusion that oestrogen produced neuroexcitatory effects, leading to increased voluntary activation at late‐follicular phase, while progesterone caused neuroinhibitory effects, resulting in greater intracortical inhibition and reduced voluntary activation at mid‐luteal phase. Smith et al. (2002) reported similar results, as they found decreased facilitation, that is, decreased motor‐evoked potential measured with transcranial magnetic stimulation (TMS) at mid‐luteal phase compared to EF phase.

Our findings indicate that sex differences in neural responses were evident not only during IMVC but also during dynamic balance perturbations when females were at LUT phase of MC. Both neural drive assessed with the V‐wave and spinal excitability measured with the H‐reflex during dynamic balance perturbations were significantly lower in females at LUT compared to males – a difference not observed at EF. The most distinct difference in these MC phases is that the LUT phase is characterized by significantly higher concentrations of E2 and P4 than EF. Especially the inhibitory role of P4 has been reported earlier (Ansdell et al., 2019; Smith et al., 2002), which can explain the significant sex differences observed at LUT in our study. In the case of the H‐reflex, direct hormonal influence on spinal excitability has not been clearly established, and thus the most explanatory factor for the decreased H‐reflex found during dynamic balance perturbations may be presynaptic inhibition. It is quite evident that in presynaptic inhibition the Ia fibres are under a strong inhibitory influence by input from descending corticospinal fibres that may downregulate the spinal excitability, that is, the H‐reflex (Baudry & Duchateau, 2014; Llewellyn et al., 1990; Tokuno et al., 2008). Previously, the influence of oestrogen in decreasing presynaptic inhibition was shown in study by Hoffman et al. (2018b), and considering the fact that in clinical rodent models the phenomenon of GABAergic inhibition has been evidenced to modulate specifically brain functions (Majewska et al., 1986; Smith et al., 1989), it may be plausible to suggest, that the central excitatory actions are downregulated at cortical level, which then modulates the spinal excitability via presynaptic inhibition.

Our results suggest that the presence or absence of sex differences in neural responses may be influenced by menstrual cycle phase, likely due to hormonal fluctuation. Specifically, the higher concentration of P4 at LUT may attenuate neural responses, thereby contributing to a meaningful sex difference. Although E2 also increases at LUT and even exceeds levels observed at EF, both our study and several MC‐related studies (Ansdell et al., 2019; Smith et al., 2002) suggest that the inhibitory effects of P4 seems to predominate at LUT. In fact, as seen in the hormonal results, a significant decrease in E2:P4 ratio was observed from EF to LUT, indicating a significant P4 dominance at LUT. As such, it seems plausible to suggest that female reproductive hormones have at least some level of influence on neural responses and that the variation and changing dominance in hormonal concentrations between MC phases may contribute to our observations of sex differences. It must be noted that several mechanisms might contribute to the neural outcomes. Factors associated with the MC, such as painful menstruation (Ferries‐Rowe et al. 2020) or elevated pain threshold, referred to as luteal analgesia (Vincent et al., 2018), are known to modulate pain sensitivity across MC and consequently influence the neural mechanisms. Furthermore, motivation and especially circadian rhythm can alter strength outcomes and may even outweigh MC effects (Beníčková et al., 2025). Although we did not measure or control these factors, except for circadian rhythm, the pattern of pain‐related mechanisms is unlikely to account for sex specific neural differences at EF and LUT phases in our study.

Females exhibited significantly higher TIB/SOL_RATIO_% than males when measurements were conducted at EF, whereas no significant sex differences were observed at LUT. The TIB/SOL_RATIO_% was used to reflect the contribution of TIB as an antagonist muscle during dynamic balance perturbations (i.e., the change in co‐activation). The agonist muscle has projections to antagonist muscle via Ia inhibitory interneurons, suppressing the activity of antagonist muscle motoneurons (Pierrot‐Deseilligny & Burke, 2005), thus changing the amount of agonist–antagonist co‐activation. This pathway is also strongly modulated by supraspinal pathways (Crone & Nielsen, 1989). By modulating the excitatory and inhibitory inputs onto Ia inhibitory interneurons, supraspinal centres can reduce reciprocal inhibition of muscles and enable increased co‐contraction, consequently controlling the relative amount of joint stiffness to meet the requirements of the ongoing motor task. The higher antagonist muscle activation suggests that females manifested lower reciprocal inhibition and increased co‐activation during dynamic balance perturbations. However, progesterone‐accompanying GABAergic inhibition at LUT influencing increased co‐activation is not the likely explanation here since females exhibited significantly greater co‐activation at EF. Indeed, it has been reported that females, in general, exhibit greater co‐activation than males during walking (Mengarelli et al., 2017), hopping (Padua et al., 2005), balancing (Khowailed & Lee, 2021) and low‐level tonic isometric contractions (Mendonca et al., 2020). Therefore, it may be plausible to suggest that the sex differences in co‐activation found in our study were not influenced by females’ MC and the fluctuation in ovarian hormones, but perhaps females in general used different control strategies for improving joint stiffness to meet the requirements of balance perturbations.

According to our results, it seems that MC phase may influence the observation of sex differences in neural responses, but not necessarily in performance outputs. In IMVC, males exhibited higher force production when compared to females at both EF and LUT, as was expected due to males’ generally larger muscle cross‐sectional area (Staron et al., 2000). Also, in dynamic balance perturbations, no MC‐related sex differences were observed. Although sex differences were evident in the COP of anterior–posterior sway, this occurred regardless of MC phase in all three measured parameters. In the COP of mediolateral sway there were no sex differences at all whether females were measured at EF or LUT. As the perturbations were induced only in the anterior–posterior direction, and only posterior sways (participant sway forward) were analysed, the role of mediolateral sway during such controlled and restricted balance perturbations may not be as crucial for restoring the balance.

### 4.1 Limitations

Although we measured a total of 19 females, the final number of participants used in statistical analysis was reduced to 10. This underlines the challenges faced when MC‐related measurements with multiple measurement points are implemented. MC tracking and the retrospective analysis of hormones helped to ensure the quality of data (Elliott‐Sale et al., 2021), but the presence of possible menstrual irregularities or missed timing of measurements resulted in inevitable dropouts.

### 4.2 Strengths

Tracking of MC was implemented in line with current recommendations for best practice (Elliott‐Sale et al., 2021). Venous blood samples were taken in the morning of the measurement day to retrospectively confirm the exact levels of E2 and P4, and participants were excluded if P4 levels at LUT were <16 nmol/l in line with the guidelines of Elliott‐Sale et al. (2021). During H‐reflex measurements in dynamic balance perturbation, the M‐wave was controlled strictly and on‐line and only trials where the M‐wave was 10 ± 3% were accepted to (1) keep the H‐reflex on the ascending portion of the recruitment curve and (2) ensure a stable M‐wave, which suggests that constant number of motor nerve fibres were excited (Knikou, 2008). This latter methodological consideration enhances the comparability of data across subjects.

### 4.3 Conclusion

During dynamic balance perturbations, both spinal excitability measured with the H‐reflex and neural drive measured with the V‐wave were significantly lower between males and females at LUT, which is characterized by elevated concentrations of endogenous sex hormones oestradiol and especially inhibitory progesterone. Similarly, the V‐wave during IMVCs was lower in females at LUT compared to males. However, MC phase and the fluctuations in ovarian hormones seemed to have no influence on balance properties. Dynamic balance measured as the COP of anterior–posterior sway was poorer and muscle co‐activation greater in females compared to males regardless of MC phase. Our findings demonstrate that MC phase and the fluctuation in ovarian hormones can influence the interpretation of sex differences in neural responses during dynamic balance perturbations. To conclude, testing females under varying hormonal milieus likely increases variance in the data, and thus it may be important to measure sex hormone concentrations when assessing sex differences to ensure normally menstruating females are in the targeted phase for the study question.

## AUTHOR CONTRIBUTIONS

The work was designed by Samuli Nevanperä, Jarmo M. Piirainen, and Ritva S. Mikkonen. Samuli Nevanperä carried out data acquisition, analysis, and drafting of the original manuscript. Statistical analysis was performed by Samuli Nevanperä, Simon Walker, and Jarmo M. Piirainen. All authors contributed to revising the work, have read and approved the final version of the manuscript and agree to be accountable for all aspects of the work in ensuring that questions related to the accuracy or integrity of any part of the work are appropriately investigated and resolved. All persons designated as authors qualify for authorship, and all those who qualify for authorship are listed.

## CONFLICT OF INTEREST

The authors declare no conflicts of interests.

## Figures and Tables

**FIGURE A1:**
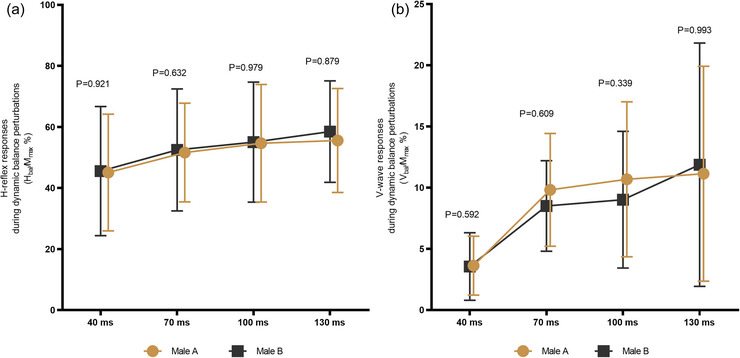
(a) The *H*_bal_/*M*_max_% between measurements A and B. No measurement × latency interaction [*F*(3, 68.199) = 0.142; *P *= 0.934] nor measurement main effect [*F*(1, 19.213) = 0.060; *P *= 0.808] was observed. A significant latency main effect was observed [*F*(3, 71.883) = 6.388; *P *< 0.001]. Pairwise comparison showed no significant differences at different latencies. (b) The *V*_bal_ *M*_max_% between measurements A and B. No measurement × latency interaction [*F*(3, 76.384) = 0.166; *P *= 0.919] nor measurement main effect [*F*(1, 32.498) = 0.899; *P *= 0.350] was observed. A significant latency main effect was obtained [*F*(3, 79.722) = 25.018; *P *< 0.001]. Pairwise comparison showed no significant differences at different latencies.

**FIGURE A2:**
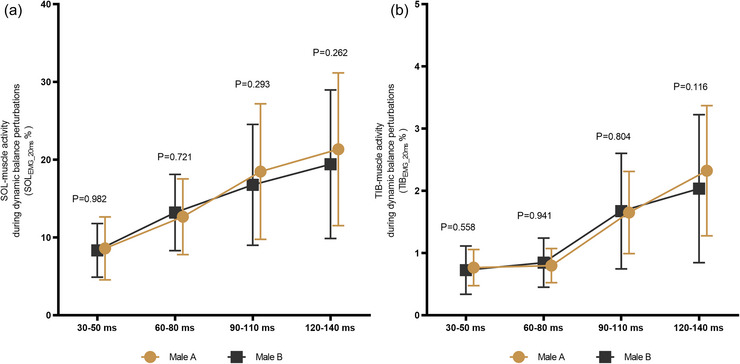
The SOL (a) and TIB (b) muscle activity during dynamic balance perturbations in 20 ms time windows coinciding with the stimulation latencies. (a) The SOL_EMG_20MS_% between measurements A and B. No measurement × latency interaction [*F*(3, 78.473) = 0.569; *P *= 0.637] nor measurement main effect [*F*(1, 32.337) = 0.592; *P *= 0.447] was observed. A significant latency main effect was observed [*F*(3, 85.960) = 42.029; *P *< 0.001]. Pairwise comparison showed no statistically significant differences at different latencies. (b) The TIB_EMG_20MS_% between measurements A and B. No measurement × latency interaction [*F*(3, 66.219) = 0.730; *P *= 0.538] nor measurement main effect [*F*(1, 21.130) = 0.809; *P *= 0.379] was observed. A significant latency main effect was observed [*F*(3, 74.924) = 61.359; *P *< 0.001]. Pairwise comparison showed no statistically significant differences at different latencies.

**FIGURE A3:**
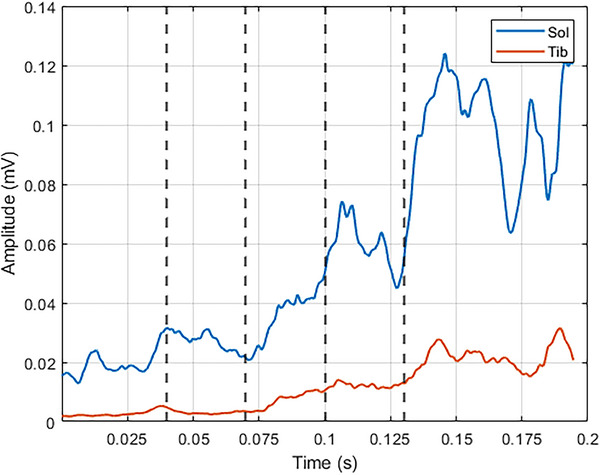
A typical raw muscle activity in millivolts (rectified and smoothed with time constant of 0.003 s in Spike2 software) of SOL (blue line) and TIB (red line) from a single male participant. The data are a waveform average of all 10 posterior perturbations induced during the dynamic balance set with no electrical stimulation and starting from the onset of ankle movement. Vertical lines depict the four stimulation latencies (40, 70, 100 and 130 ms).

**FIGURE A4:**
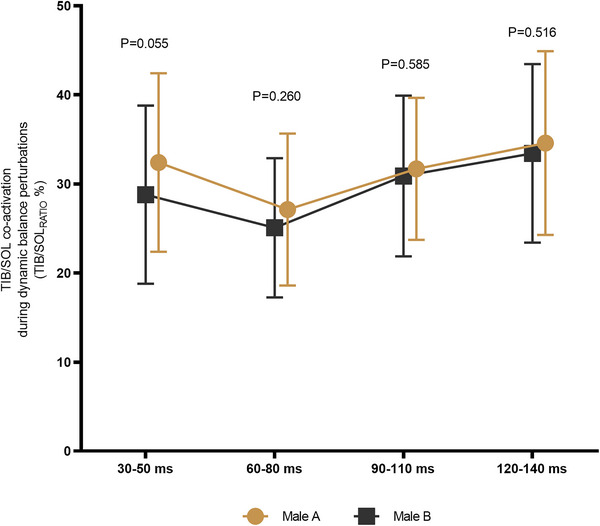
The TIB/SOL_RATIO_% between measurements A and B. No measurement × latency interaction [*F*(3, 61.194) = 0.391; *P *= 0.760] or measurement main effect [*F*(1, 18.788) = 2.438; *P *= 0.135] was observed, but a significant latency main effect [*F*(3, 67.917) = 11.456; *P *< 0.001] was obtained. Pairwise comparison showed no significant differences at different latencies.

## Data Availability

The gathered data of this study are available from the corresponding author on reasonable request.

## 1

Here we provide the test–retest analysis of males’ first (A) and second (B) measurements, confirming measurement stability and absence of variability.

From dynamic balance perturbations, the H‐reflex (Figure A1a), V‐wave (Figure A1b), SOL EMG activity (Figure A2a), TIB EMG activity (Figure A2b), a typical raw muscle activity of SOL and TIB (Figure A3) and TIB/SOL_RATIO_% (Figure A4) are presented, as well as the intraclass correlation of H‐reflex and V‐wave measurements at different latencies (Tables A1 and A2, respectively). Table A3 shows the data obtained from isometric maximal voluntary contractions and dynamic balance from anterior–posterior sway and mediolateral sway and the concentration of total testosterone (tTesto). The same statistical methods (linear mixed model) were used as presented in the main text. To analyse the test–retest reliability and consistency of measurements for main variables (*H*_bal_/*M*_max_% and *V*_bal_/*M*_max_%), the intraclass correlation coefficient was used (reported in Tables A1 and A2, respectively). The level of significance was set at *P *= 0.05.

**TABLE A1:** *H*_bal_/*M*_max_% intraclass correlation at each latency.

Delay	ICC	95% CI	*P*	Correlation
40 ms	0.797	0.396–0.932	0.003	Moderate
70 ms	0.777	0.335–0.925	0.004	Moderate
100 ms	0.669	0.053–0.884	0.020	Moderate
130 ms	0.798	0.421–0.929	0.002	Moderate

Correlations: ICC 0.11–0.40 = slight, ICC 0.41–0.60 = fair, ICC 0.61–0.80 = moderate, ICC 0.81–1.0 = substantial (Mendonca et al. 2019; Solstad et al. 2011). Abbreviations: ICC, intraclass correlation coefficient; CI, confidence interval.

**TABLE A2:** *V*_bal_/*M*_max_% intraclass correlation at each latency.

Delay	ICC	95% CI	*P*	Correlation
40 ms	0.752	0.291–0.913	0.005	Moderate
70 ms	0.791	0.401–0.927	0.002	Moderate
100 ms	0.923	0.779–0.973	<0.001	Substantial
130 ms	0.916	0.758–0.970	<0.001	Substantial

Correlations: ICC 0.11–0.40 = slight, ICC 0.41–0.60 = fair, ICC 0.61–0.80 = moderate, ICC 0.81–1.0 = substantial (Mendonca et al. 2019; Solstad et al. 2011). Abbreviations: ICC, intraclass correlation coeffcient; CI, confidence interval.

**TABLE A3:** Comparison between males’ measurements A and B for tTesto and performance parameters from isometric maximal voluntary contraction of plantar flexion as well as dynamic balance from anterior–posterior sway and mediolateral sway.

Variable	Male A	Male B	*P*
tTesto (nmol/l)	15.93 ± 2.37	16.83 ± 4.18	0.272
Isometric maximal voluntary contraction			
MVC (N)	2239.5 ± 367.8	2335.4 ± 373.2	0.088
*V*/*M*_max_%	60.2 ± 21.5	54.7 ± 20.0	0.118
Dynamic balance parameters			
Anterior–posterior direction sway			
COP_p‐to‐p_ (mm/(weight × height))	0.925 ± 0.201	0.950 ± 0.261	0.469
COP_velocity_ (mm s^−^^1^/(weight × height))	1.134 ± 0.280	1.144 ± 0.372	0.846
COP_total trace_ (mm/(weight × height))	1.866 ± 0.435	1.779 ± 0.570	0.376
Mediolateral direction sway			
COP_p‐to‐p_ (mm/(weight × height))	0.773 ± 0.567	0.744 ± 0.736	0720
COP_velocity_ (mm s^−^^1^/(weight × height))	1.159 ± 0.979	1.114 ± 1.192	0.760
COP_total trace_ (mm/(weight × height))	1.750 ± 1.377	1.681 ± 1.841	0.792

*Note*: Data are means ± SD. All values of dynamic balance parameter are normalized to weight × height. Level of significance set at *P *< 0.05. Abbreviations: IMVC, isometric maximal voluntary contraction of plantar flexion; COP_p‐to‐p_, centre of pressure in peak‐to‐peak amplitude; COP_velocity_, centre of pressure in velocity; COP_total trace_, centre of pressure in total sway.
